# Caucasian Ethnicity, but Not Treatment Cessation Is Associated with HBsAg Loss Following Nucleos(t)ide Analogue-Induced HBeAg Seroconversion

**DOI:** 10.3390/v11080687

**Published:** 2019-07-26

**Authors:** Stijn Van Hees, Heng Chi, Bettina Hansen, Stefan Bourgeois, Hans Van Vlierberghe, Thomas Sersté, Sven Francque, David Wong, Dirk Sprengers, Christophe Moreno, Frederik Nevens, Harry Janssen, Thomas Vanwolleghem

**Affiliations:** 1 Department of Gastroenterology and Hepatology, Antwerp University Hospital, 2650 Antwerp, Belgium; 2 Laboratory of Experimental Medicine and Pediatrics, University of Antwerp, 2650 Antwerp, Belgium; 3 Department of Gastroenterology and Hepatology, Erasmus MC University Medical Center Rotterdam, 3015 AA Rotterdam, The Netherlands; 4 Toronto Centre of Liver Disease, University Health Network, Toronto General Hospital, University of Toronto, Toronto, ON M5G2C4, Canada; 5 Department of Gastroenterology and Hepatology, ZNA Stuivenberg, 2060 Antwerp, Belgium; 6 Department of Gastroenterology and Hepatology, Ghent University Hospital, 9000 Ghent, Belgium; 7 Department of Gastroenterology and Hepatology, Saint-Pierre University Hospital, 1000 Brussels, Belgium; 8 Department of Gastroenterology and Hepatology, GZA Antwerp, 2610 Antwerp, Belgium; 9 Department of Gastroenterology, Hepatopancreatology and Digestive Oncology, CUB Hôpital Erasme, Université Libre de Bruxelles, 1050 Brussels, Belgium; 10 Department of Hepatology, University Hospitals KULeuven, 3000 Leuven, Belgium

**Keywords:** chronic hepatitis B, treatment cessation, HBsAg seroclearance, ethnicity

## Abstract

It is well appreciated that ethnicity influences the natural history and immune responses during a chronic hepatitis B infection. In this study, we explore the effect of ethnicity and treatment cessation on Hepatitis B surface Antigen (HBsAg) seroclearance in patients with Nucleos(t)ide Analogue (NA)-induced Hepatitis B e Antigen (HBeAg) seroconversion. We performed a multi-ethnic, multicentric observational cohort study. The analyzed cohort consisted of 178 mono-infected, predominantly male (75.3%) chronic hepatitis B patients of mixed ethnicity (44.4% Asians, 48.9% Caucasians) with nucleos(t)ide analogue-induced HBeAg seroconversion. Treatment was withdrawn in 105 patients and continued in 73, leading to HBsAg loss in 14 patients off- and 16 patients on-treatment, respectively. Overall, HBsAg loss rates were not affected by treatment cessation (hazard ratio 1.45, *p* = 0.372), regardless of consolidation treatment duration. Caucasian ethnicity was associated with an increased chance of HBsAg loss (hazard ratio 6.70, *p* = 0.001), but hepatitis B virus genotype was not (*p* = 0.812). In conclusion, ethnicity is the most important determinant for HBsAg loss after NA-induced HBeAg seroconversion, with up to six-fold higher HBsAg loss rates in Caucasians compared to Asians, irrespective of treatment cessation and consolidation treatment duration.

## 1. Introduction

With approximately 3% of the worldwide population infected, chronic hepatitis B (CHB) represents a serious global health problem. Chronic infections are associated with an up to 100-fold increased risk for liver-related complications, including cirrhosis, decompensation, and hepatocellular carcinoma (HCC), ultimately resulting in liver-related death [1]. As such, the hepatitis B virus (HBV) continues to kill over 700,000 people annually [2].

Curing CHB is challenging. HBV integrates into the host genome and forms covalently closed circular DNA (cccDNA), a minichromosome in the nucleus of infected hepatocytes, which cannot be eradicated with the current therapeutic armamentarium [3]. However, seroclearance of the virus from blood, marked by hepatitis B surface antigen (HBsAg) loss, confers excellent clinical outcomes and is therefore regarded as the optimal endpoint in CHB treatment [4,5,6,7].

Nucleos(t)ide analogues (NA), the first line HBV antivirals, efficiently suppress viral replication, but HBsAg loss is rarely observed and is estimated to require an average of 36 to 52 years of continuous treatment [8,9]. Over the last years, withdrawal of NA treatment in long-term virally suppressed patients that were Hepatitis B e Antigen (HBeAg) negative at the start of treatment has been put forward as a strategy to increase HBsAg loss rates [3]. Several independent, observational cohort studies demonstrated high HBsAg loss rates after treatment cessation in these patients, reaching up to 39% at six years after treatment cessation in Caucasian patients versus a mere 16% in Asian patients [10,11,12,13,14]. These studies mainly focused on the outcome after treatment cessation in one country, including either predominantly Caucasian or predominantly Asian patients and were thus not powered to study the influence of ethnicity on HBsAg seroclearance [10,11,12,13,14]. Nevertheless, it is well appreciated that ethnicity influences immune responses during a chronic hepatitis B infection [15,16].

In a multi-ethnic cohort of start-of-treatment HBeAg-positive patients who discontinued treatment following NA-induced HBeAg seroconversion, we recently observed six-fold higher HBsAg loss rates in Caucasian compared to non-Caucasian patients [17]. However, little is known on the impact of treatment cessation on HBsAg seroclearance in these patients. Therefore, in the current study, we expanded our cohort with consecutive patients who continued treatment following HBeAg seroconversion and explored the relative impact of ethnicity and NA withdrawal on HBsAg seroclearance. We observed that Caucasian ethnicity is the most important determinant for HBsAg loss after NA-induced HBeAg-seroconversion, with up to six-fold higher HBsAg loss rates in Caucasians compared to Asians, irrespective of treatment cessation and consolidation treatment duration.

## 2. Patients and Methods

### 2.1. Study Design

We previously established a retrospective, observational cohort of CHB patients (*n* = 98) who discontinued treatment following NA-induced HBeAg seroconversion at 20 hospitals in Belgium (*n* = 18), the Netherlands (*n* = 1), and Canada (*n* = 1) between 1998 and 2016 [17]. We now expanded this cohort with consecutive patients recruited from all participating centers which continued NA treatment following treatment-induced HBeAg seroconversion until (1) the end of study inclusion (December 2016), (2) lost to follow-up or (3) HBsAg seroclearance using the same exclusion criteria, definitions and data collection strategy as described before [17]. Following HBeAg seroconversion, treatment was continued or interrupted at the discretion of the treating physician, according to the prevailing guidelines or reimbursement criteria in the different countries. Canadian treatment guidelines state that treatment cessation may be considered following HBeAg seroconversion with a minimum of 12 months consolidation treatment [18]. Dutch guidelines recommend continuation of antiviral therapy until HBsAg loss [19]. Belgian reimbursement guidelines impose the discontinuation of treatment following HBeAg seroconversion [20]. Ethical approval was obtained with the committee for medical ethics of the Antwerp University Hospital as central medical ethical committee (reference number: 15/44/464). Written informed consent was not deemed necessary by the medical ethical committee given the retrospective character of the study with inclusion of patients over a long period of time (1998–2016) and a substantial proportion of the patients that were loss to follow-up or dead by the time of inclusion.

### 2.2. Statistical Analysis

Continuous variables are shown as mean ± standard deviation (SD) or median (interquartile range (IQR)). Variables were compared between groups using a chi-square test for categorical variables and a Mann–Whitney *U*-test or Student *t*-test for continuous variables. Consolidation treatment duration was calculated as the time from HBeAg seroconversion until treatment cessation.

A Cox regression model was used to investigate factors associated with HBsAg loss following HBeAg seroconversion. This approach allows for correction of (1) the time it takes for HBsAg loss to occur after HBeAg seroconversion, and (2) inter-patient differences in follow-up time. Follow-up time was calculated as the time from HBeAg seroconversion until HBsAg loss, retreatment initiation or loss to follow-up. Treatment cessation was included as a time-dependent covariate since patients in the treatment cessation group discontinued treatment at a variable point in time after HBeAg seroconversion [21]. Variables with *p* < 0.100 in univariate analysis were further investigated in multivariate analyses. To account for possible heterogeneity between Belgian and non-Belgian centers, Cox regression analyses were stratified by treatment country. A clock-reset approach was applied to plot Kaplan–Meier curves to illustrate the differences in HBsAg loss rates between patients under continuous treatment and patients who discontinued treatment [22,23,24]. Patients who discontinued treatment were censored in the continuous treatment group at the time of treatment interruption. The “clock” of these patients was then reset to zero at the time of treatment interruption and further follow-up of these patients was plotted in the treatment cessation group. Data were analyzed in SPPS version 24.0 (IBM, Armonk, NY, USA). All statistical tests were two-sided and *p*-values < 0.05 were considered statistically significant.

## 3. Results

### 3.1. Cohort Characteristics

**Figure 1:**
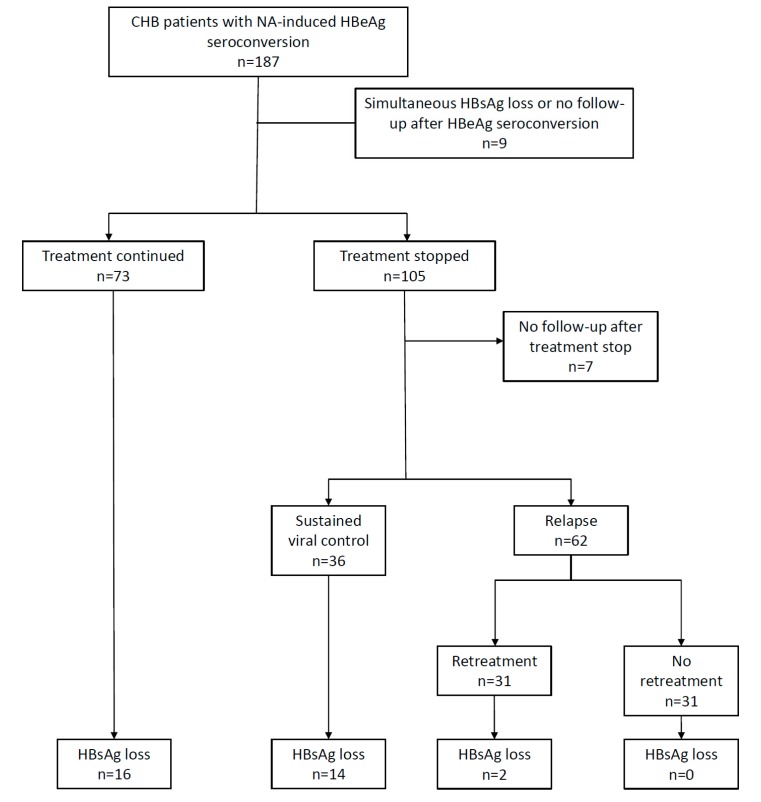
Flowchart of the study cohort according to treatment modality and virologic outcome. NA: Nucleos(t)ide Analogues, HBsAg: Hepatitis B surface antigen, HBeAg: Hepatitis B e antigen, CHB: chronic hepatitis B. Relapse was defined as a single HBV DNA elevation >2000 IU/mL after treatment cessation.

After expansion of the NA stop cohort with patients who continued treatment, the final cohort consisted of 178 predominantly male (75.3%) patients of well-balanced, mixed ethnicity (44.4% Asians, 48.9% Caucasians) with NA-induced HBeAg seroconversion after a median treatment duration of 15.5 (8.2–29.3) months [17]. Following HBeAg seroconversion, 105 patients continued treatment for a median of 11.4 (6.1–18.0) months before withdrawal, whereas 73 patients continued treatment until the end of study inclusion, loss to follow-up or HBsAg loss. Seven patients were lost to follow-up immediately after treatment withdrawal (Figure 1). Patient characteristics per treatment status after HBeAg seroconversion are depicted in Table 1. Overall, characteristics were similar between both groups. However, the group continuing treatment consisted of significantly more start-of-treatment cirrhotic patients (41.1% vs. 22.8%, *p* = 0.012), was treated more frequently with a second-generation NA (83.5% vs. 41.0%, *p* < 0.001) and had a significantly shorter follow-up time after HBeAg seroconversion (median 22.2 vs. 64.2 months, *p* < 0.001) compared to the treatment stop group.

### 3.2. HBsAg Loss Following HBeAg Seroconversion

Thirty patients presented with HBsAg loss at a median of 15.2 (5.2–23.8) months after HBeAg seroconversion: 16 on-treatment after a median consolidation therapy of 5.8 (3.3–16.5) months and 14 off-treatment at a median of 9.7 (5.3–20.7) months after treatment cessation. Virologic outcomes after treatment cessation have been described elsewhere [17]. A summary is depicted in Figure 1.

### 3.3. Caucasian Ethnicity, but not Treatment Cessation Is Associated with HBsAg Loss

**Figure 2:**
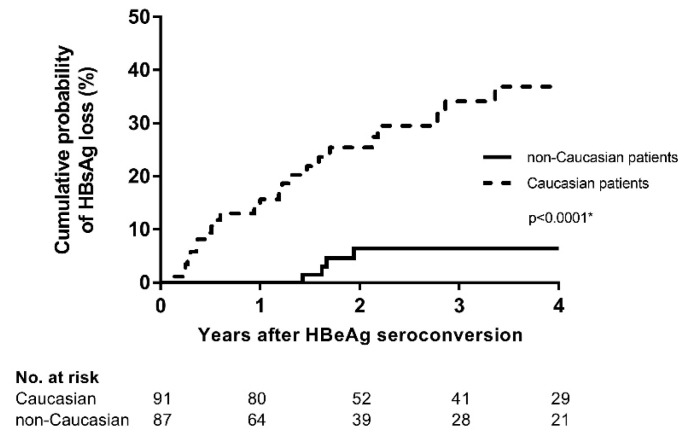
Kaplan–Meier curve showing the difference in HBsAg loss rates between patients of Caucasian and non-Caucasian ethnicity irrespective of treatment status after HBeAg seroconversion. * *p*-value = log-rank. HBsAg: Hepatitis B surface Antigen, HBeAg: Hepatitis B e Antigen.

Factors assessed to predict HBsAg loss after HBeAg seroconversion in the entire cohort are displayed in Table 2. Caucasian ethnicity (Hazard Ratio (HR) 6.70, *p* = 0.001), higher HBV DNA (HR 2.29 per log increment, *p* < 0.001), higher gamma-GT (HR 1.02 per 10 units increment, *p* = 0.006) and older age at start of treatment (HR 1.03 per year increment, *p* = 0.006) were associated with HBsAg loss in a univariate cox regression model. In multivariate analyses, Caucasian ethnicity (Figure 2 and Table 2) and higher HBV DNA levels at the start of treatment continued to be significantly associated (Table 2). The HBV genotype was not associated with HBsAg loss (*p* = 0.812) in a subpopulation of 48 patients in which HBV genotyping was available.

**Figure 3:**
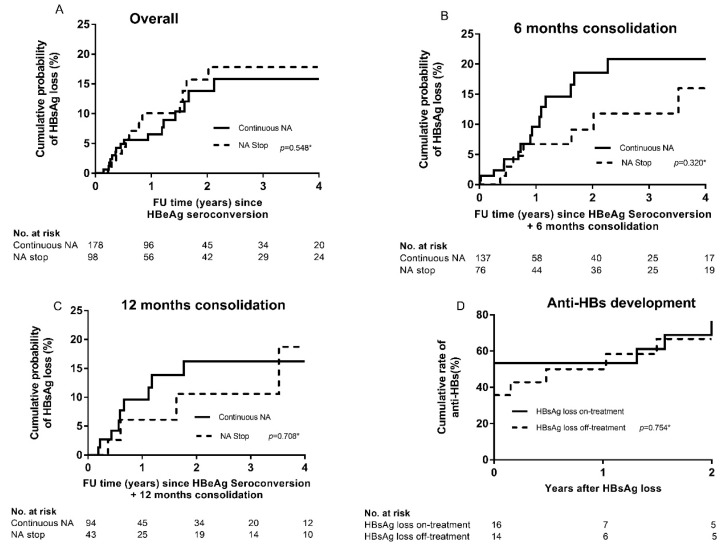
Kaplan–Meier curves showing (1) the cumulative probability of HBsAg loss after HBeAg seroconversion using a clock-reset approach (**A**–**C**) and (2) the cumulative probability of anti-HBs development after HBsAg loss (**D**). Analysis of (**A**) the entire cohort. Origin of X-axis is set at HBeAg seroconversion. (**B**) Patients with at least six months consolidation treatment. Origin of X-axis is set at six months after HBeAg seroconversion. (**C**) Patients with at least 12 months consolidation treatment. Origin of X-axis is set at 12 months after HBeAg seroconversion and (**D**) all patients with HBsAg loss. As described in the Methods section, patients in the continuous treatment group were censored at the time of HBsAg loss, loss to follow-up or treatment cessation. Patients in the treatment cessation group were censored at the time of HBsAg loss, loss to follow-up or retreatment initiation. Patients were considered continuing treatment until treatment was discontinued and were censored in the group continuing treatment at the time of treatment cessation. The moment of treatment cessation was subsequently considered as time zero for further follow-up in the treatment stop group. * *p*-value = log-rank, HBeAg: Hepatitis B e antigen, HBsAg: Hepatitis B surface antigen, NA: Nucleos(t)ide Analogues, Anti-HBs: Anti hepatitis B surface antigen antibody. FU: Follow-Up.

Overall, mean annual HBsAg loss rates were 5.4% and 5.1% on- and off-treatment respectively (*p* = 0.561). In Cox regression analyses, treatment cessation was not associated with HBsAg loss (HR 1.45, *p* = 0.372, Table 2, Figure 3A). Prolonging treatment duration following HBeAg seroconversion to at least 6 (HR 1.73, *p* = 0.289, Figure 3B) or 12 months (HR 1.52, *p* = 0.525 Figure 3C) before cessation, did not result in higher HBsAg loss rates compared to patients that continued antiviral treatment. Anti-HBs antibody (Anti-HBs) development was observed in 12 patients on- and seven patients off-treatment. Patients with on-treatment HBsAg loss tended to develop anti-HBs more rapidly, but there was no difference in the cumulative rate of anti-HBs development two years after HBsAg loss (68.9% vs. 66.6%, *p* = 0.754) (Figure 3D).

To control for a possible imbalance in our cohort regarding cirrhosis status and type of NA use, we performed a propensity score-adjusted Cox regression analysis. Propensity scores were derived from a logistic regression model assessing the correlation between cirrhosis status, NA type, and (1) Caucasian ethnicity or (2) treatment cessation. Results showed that propensity score adjustment did not change our observations (Table S1), indicating that the imbalance in cirrhosis status and type of NA did not impact our results.

## 4. Discussion

In the present study, we investigated HBsAg loss following NA-induced HBeAg seroconversion in a multicentric, international CHB cohort that was well-balanced for ethnicity. We demonstrate that Caucasian ethnicity, but not treatment cessation is associated with HBsAg loss.

In contrast to previous reports in start-of-treatment HBeAg-negative patients, treatment cessation did not increase the chance of subsequent HBsAg loss in our cohort. In long-term virally suppressed, start-of-treatment HBeAg-negative patients, treatment cessation has been suggested to reinvigorate immune responses leading to subsequent HBsAg loss [13]. HBeAg seroconversion marks improved immune control [3]. As such, patients with a recent NA-induced HBeAg seroconversion may be prone to clear HBsAg through this improved immune control, regardless of treatment cessation.

Interestingly, irrespective of treatment cessation, Caucasian patients had a >6-fold increased chance of HBsAg loss compared to other ethnicities, which comprised almost half of the entire cohort. Previous studies corroborate an up to 2.8-fold lower chance of HBsAg loss in Asian patients during the natural history of a CHB infection [25,26]. In a subgroup (*n* = 48) of our cohort with an available HBV genotype, this ethnic difference in HBsAg loss rates was not related to the HBV genotype. Host factors such as Human Leukocyte Antigen (HLA) polymorphisms may evidently contribute to the observed differences in HBsAg loss rates [27]. Polymorphisms of Major Histocompatibility Complex class II proteins, have been shown to be associated with HBV persistence, seroconversion, seroclearance, and disease progression, but only in patients with a certain ethnic background. For example, the HLA-DR polymorphism rs 9277535 (550 A/G) is strongly associated with chronic hepatitis B and its outcomes in Asian, but not in African-American or Caucasian patients [15,16].

Although this is, to our knowledge, the largest real-life, multi-ethnic cohort study as of today assessing HBsAg loss in patients with NA treatment-induced HBeAg seroconversion, still there are some limitations. First, the study design was intrinsically retrospective. As such, a variation in the number of lab values available per patient cannot be overcome and the duration of infection was mostly unknown. In addition, HBV genotypes were available in only 48 patients as HBV genotyping was mostly not performed during routine clinical practice. Nonetheless, most patients were followed-up every three to six months according to international guidelines and the age at the start of treatment was not associated with HBsAg loss in multivariate modelling. Second, we pooled patients from 20 centres in Belgium, the Netherlands, and Canada over a long period of time (1998–2016), possibly resulting in a heterogeneous study population. Nevertheless, data were collected in a systematic manner in all centres and correction of the statistical analyses for possible heterogeneity did not influence the results. Third, the group continuing treatment contained significantly more cirrhotic patients, was more frequently treated with second-generation NA and had a shorter follow-up time. However, because (1) cirrhosis and the type of NA were not associated with HBsAg loss in our Cox regression model, (2) propensity score-adjusted Cox regression analysis to correct for differences in NA type and cirrhosis status yielded similar results (Table S1), and (3) Cox regression analyses and Kaplan–Meier curves inherently correct for a difference in follow-up time, we are convinced that this unbalance did not impact our results.

In conclusion, ethnicity is the most important determinant for HBsAg loss after NA-induced HBeAg seroconversion, with an up to six-fold increased chance of HBsAg loss in Caucasian compared to non-Caucasian patients, irrespective of treatment cessation and consolidation treatment duration. Further prospective studies should unravel the immunological events in order to improve functional cure rates of future HBV antivirals.

## Figures and Tables

**Table 1:** Patient characteristics according to treatment status after HBeAg seroconversion. *: HBV Genotyping was available in 48 patients, NA: Nucleos(t)ide analog, ULN: Upper limit of normal, ALT: Alanine amino transferase, °: Mean ± SD, °°: Median (IQR), FU: Follow-up. Significant *p*-values (<0.05) are marked in bold.

		NA Stop	Continuing NA	*p*-Value
		*n* = 105	*n* = 73	
Baseline characteristics	Male gender	78 (74%)	56 (77%)	*0.713*
	Age at treatment start °	40.2 ± 15.2	41.4 ± 15.0	*0.484*
	*Ethnicity*			*0.874*
	Asian (n)	46	33	
	Black (n)	7	5	
	Caucasian (n)	52	34	
	Other (n)		1	
	Cirrhosis (n)	24 (22.8%)	30 (41.1%)	***0.012***
	Previous interferon treatment (n)	24 (22.6%)	19 (26.4%)	*0.722*
	HBV genotype *	7A, 1B, 7C, 3D	10A, 4B, 10C, 5D, 1G	*0.836*
Treatment start	HBV DNA (^10^log) °	6.86 ± 1.62	7.13 ± 1.85	*0.296*
	ALT (ULN) °°	2.9 (2.0–5.4)	3.7 (1.7–7.0)	*0.728*
	Year of treatment start °°	2006 (2003–2010)	2010 (2007–2012)	***<0.001***
HBeAg seroconversion	HBV DNA (^10^log) °	1.75 ± 0.73	1.85 ± 1.08	*0.588*
	ALT (ULN) °°	0.8 (0.5–1.0)	0.8 (0.6-1.2)	*0.230*
	*Type NA*			***<0.001***
	First generation (Lamivudine-Adefovir-Telbivudine)	62	12	
	Second generation (Entecavir-Tenofovir)	43	61	
	Time to HBeAg seroconversion (months) °°	17.8 (8.4–31.9)	14.2 (6.0–26.4)	*0.611*
	FU-time after HBeAg seroconversion (months) °°	64.9 (34.0–97.2)	22.2 (12.0–42.1)	***<0.001***
	Consolidation treatment (months) °°	11.4 (6.1–18.0)	NA	*NA*
	FU-time after treatment stop (months) °°	39.8 (13.7–81.4)	NA	*NA*

**Table 2:** Univariate and multivariate Cox regression model to predict HBsAg loss following treatment-induced HBeAg seroconversion in the entire patient cohort. HR: Hazard ratio, C.I.: Confidence interval, ALT: Alanine aminotransferase, AST: Aspartate aminotransferase, Gamma-GT: Gamma-glutamyl transferase. HBsAg: Hepatitis B surface Antigen, HBeAg: Hepatitis B e Antigen. Factors with *p* < 0.100 in univariate analysis were further investigated in multivariate models. Treatment cessation was investigated as a time-dependent covariate.

		Univariate		Multivariate Model 1		Multivariate Model 2		Multivariate Model 3		Multivariate Model 4	
		*p*	HR (95% C.I.)	*p*	HR (95% C.I.)	*p*	HR (95% C.I.)	*p*	HR (95% C.I.)	*p*	HR (95% C.I.)
Baseline characteristics	Caucasian ethnicity	*0.001*	6.70 (2.26–21.40)	*0.032*	3.79 (1.12–12.77)	*0.047*	8.09 (1.03–63.81)	*0.008*	6.03 (1.59–22.84)	*0.006*	5.25 (1.62–17.03)
	Male gender	*0.198*	2.01 (0.70–5.87)								
	Prior interferon treatment	*0.724*	1.16 (0.51–2.62)								
	Cirrhosis	*0.192*	0.61 (0.28–1.29)								
Treatment start	Age at start of treatment (years)	*0.006*	1.03 (1.01–1.05)	*0.095*	1.02 (1.00–1.05)	*0.169*	1.02 (0.99–1.05)			*0.129*	1.02 (1.00–1.04)
	ALT (per 10 units increment)	*0.724*	1.00 (0.99–1.01)								
	AST (per 10 units increment)	*0.475*	1.00 (0.99–1.02)								
	Gamma-GT (per 10 units increment)	*0.006*	1.02 (1.01–1.04)			*0.091*	1.02 (1.00–1.03)				
	HBV DNA (^10^log)	*<0.001*	2.29 (1.60–3.27)	*<0.001*	1.90 (1.36–2.66)						
	Platelets (per 10^4^ units increment)	*0.989*	1.00 (0.94–1.06)								
HBeAg seroconversion	ALT (per 10 units increment)	*0.005*	1.07 (1.02–1.13)					*0.141*	1.06 (0.98–1.13)	*0.028*	1.06 (1.01–1.11)
	AST (per 10 units increment)	*0.432*	1.07 (0.91–1.26)								
	Gamma-GT (per 10 units increment)	*0.106*	1.04 (0.99–1.09)								
	HBV DNA (^10^log)	*0.059*	1.63 (0.98–2.70)					*0.751*	1.11 (0.59–2.07)		
	Platelets (per 10^4^ units increment)	*0.273*	1.03 (0.98–1.09)								
	Time to HBV DNA <2000 IU/mL (years)	*0.998*	1.00 (0.74–1.35)								
	Time to HBeAg seroconversion (years)	*0.381*	0.91 (0.73–1.13)								
	First/second generation NA	*0.823*	1.09 (0.51–2.33)								
Treatment cessation	Overall (Yes/No)	*0.372*	1.45 (0.64–3.29)								
	At least 6 months consolidation	*0.289*	1.73 (0.63–4.79)								
	At least 12 months consolidation	*0.525*	1.52 (0.42–5.47)								

## Supplementary Materials

The following are available online at https://www.mdpi.com/1999-4915/11/8/687/s1, Table S1: Propensity score adjusted Cox regression analysis to predict HBsAg loss following treatment-induced HBeAg seroconversion in the entire patient cohort.

## References

[1] Seto W.K., Lo Y.R., Pawlotsky J.M., Yuen M.F. (2018). Chronic hepatitis B virus infection. Lancet (Lond. Engl.).

[2] Stanaway J.D., Flaxman A.D., Naghavi M., Fitzmaurice C., Vos T., Abubakar I., Abu-Raddad L.J., Assadi R., Bhala N., Cowie B. (2016). The global burden of viral hepatitis from 1990 to 2013: Findings from the Global Burden of Disease Study 2013. Lancet (Lond. Engl.).

[3] Lampertico P., Agarwal K., Berg T., Buti M., Janssen H., Papatheodoridis G., Zoulim F., Tacke F. (2017). EASL 2017 Clinical Practice Guidelines on the management of hepatitis B virus infection. J. Hepatol..

[4] Chen Y.C., Sheen I.S., Chu C.M., Liaw Y.F. (2002). Prognosis following spontaneous HBsAg seroclearance in chronic hepatitis B patients with or without concurrent infection. Gastroenterology.

[5] Kim G.A., Lim Y.S., An J., Lee D., Shim J.H., Kim K.M., Lee H.C., Chung Y.H., Lee Y.S., Suh D.J. (2014). HBsAg seroclearance after nucleoside analogue therapy in patients with chronic hepatitis B: Clinical outcomes and durability. Gut.

[6] Yang H.I., Lu S.N., Liaw Y.F., You S.L., Sun C.A., Wang L.Y., Hsiao C.K., Chen P.J., Chen D.S., Chen C.J. (2002). Hepatitis B e antigen and the risk of hepatocellular carcinoma. N. Engl. J. Med..

[7] Chi H., Wong D., Peng J., Cao J., Van Hees S., Vanwolleghem T., Qi X., Chen L., Feld J.J., de Knegt R.J. (2017). Durability of Response After Hepatitis B Surface Antigen Seroclearance During Nucleos(t)ide Analogue Treatment in a Multiethnic Cohort of Chronic Hepatitis B Patients: Results After Treatment Cessation. Clin. Infect. Dis. Off. Publ. Infect. Dis. Soc. Am..

[8] Zoutendijk R., Hansen B.E., van Vuuren A.J., Boucher C.A., Janssen H.L. (2011). Serum HBsAg decline during long-term potent nucleos(t)ide analogue therapy for chronic hepatitis B and prediction of HBsAg loss. J. Infect. Dis..

[9] Chevaliez S., Hezode C., Bahrami S., Grare M., Pawlotsky J.M. (2013). Long-term hepatitis B surface antigen (HBsAg) kinetics during nucleoside/nucleotide analogue therapy: Finite treatment duration unlikely. J. Hepatol..

[10] Jeng W.J., Chen Y.C., Chien R.N., Sheen I.S., Liaw Y.F. (2018). Incidence and predictors of hepatitis B surface antigen seroclearance after cessation of nucleos(t)ide analogue therapy in hepatitis B e antigen-negative chronic hepatitis B. Hepatol. (Baltim. Md.).

[11] Hadziyannis S.J., Sevastianos V., Rapti I., Vassilopoulos D., Hadziyannis E. (2012). Sustained responses and loss of HBsAg in HBeAg-negative patients with chronic hepatitis B who stop long-term treatment with adefovir. Gastroenterology.

[12] Hung C.H., Wang J.H., Lu S.N., Hu T.H., Lee C.M., Chen C.H. (2017). Hepatitis B surface antigen loss and clinical outcomes between HBeAg-negative cirrhosis patients who discontinued or continued nucleoside analogue therapy. J. Viral Hepat..

[13] Van Bommel F., Berg T. (2018). Stopping long-term treatment with nucleos(t)ide analogues is a favourable option for selected patients with HBeAg-negative chronic hepatitis B. Liver Int. Off. J. Int. Assoc. Study Liver.

[14] Rinker F., Zimmer C.L., Honer Zu Siederdissen C., Manns M.P., Kraft A.R.M., Wedemeyer H., Bjorkstrom N.K., Cornberg M. (2018). Hepatitis B virus-specific T cell responses after stopping nucleos(t)ide analogue therapy in HBeAg-negative chronic hepatitis B. J. Hepatol..

[15] Wang L., Zou Z.Q., Wang K. (2016). Clinical Relevance of HLA Gene Variants in HBV Infection. J. Immunol. Res..

[16] Thomas R., Thio C.L., Apps R., Qi Y., Gao X., Marti D., Stein J.L., Soderberg K.A., Moody M.A., Goedert J.J. (2012). A novel variant marking HLA-DP expression levels predicts recovery from hepatitis B virus infection. J. Virol..

[17] Van Hees S., Chi H., Hansen B., Bourgeois S., van Vlierberghe H., Serste T., Francque S., Wong D., Sprengers D., Moreno C. (2019). Sustained off-treatment viral control is associated with high hepatitis B surface antigen seroclearance rates in Caucasian patients with nucleos(t)ide analogue-induced HBeAg seroconversion. J. Viral Hepat..

[18] Coffin C.S., Fung S.K., Alvarez F., Cooper C.L., Doucette K.E., Fournier C., Kelly E., Ko H.H., Ma M.M., Martin S.R. (2018). Management of Hepatitis B Virus Infection: 2018 Guidelines from the Canadian Association for the Study of Liver Disease and Association of Medical Microbiology and Infectious Disease Canada. Can. Liver J..

[19] Buster E.H., Baak B.C., Bakker C.M., Beuers U.H., Brouwer J.T., Drenth J.P., van Erpecum K.J., van Hoek B., Honkoop P., Kerbert-Dreteler M.J. (2012). The 2012 revised Dutch national guidelines for the treatment of chronic hepatitis B virus infection. Neth. J. Med..

[20] Van Hees S., Bourgeois S., van Vlierberghe H., Serste T., Francque S., Michielsen P., Sprengers D., Reynaert H., Henrion J., Negrin Dastis S. (2018). Stopping nucleos(t)ide analogue treatment in Caucasian hepatitis B patients after HBeAg seroconversion is associated with high relapse rates and fatal outcomes. Aliment. Pharmacol. Ther..

[21] Fisher L.D., Lin D.Y. (1999). Time-dependent covariates in the Cox proportional-hazards regression model. Annu. Rev. Public Health.

[22] Van der Meer A.J., Veldt B.J., Feld J.J., Wedemeyer H., Dufour J.F., Lammert F., Duarte-Rojo A., Heathcote E.J., Manns M.P., Kuske L. (2012). Association between sustained virological response and all-cause mortality among patients with chronic hepatitis C and advanced hepatic fibrosis. JAMA.

[23] Westreich D., Cole S.R., Tien P.C., Chmiel J.S., Kingsley L., Funk M.J., Anastos K., Jacobson L.P. (2010). Time scale and adjusted survival curves for marginal structural cox models. Am. J. Epidemiol..

[24] Eulenburg C., Mahner S., Woelber L., Wegscheider K. (2015). A systematic model specification procedure for an illness-death model without recovery. PLoS ONE.

[25] Lim T.H., Gane E., Moyes C., Borman B., Cunningham C. (2016). HBsAg loss in a New Zealand community study with 28-year follow-up: Rates, predictors and long-term outcomes. Hepatol. Int..

[26] Nguyen L.H., Hoang J., Nguyen N.H., Vu V.D., Wang C., Trinh H.N., Li J., Zhang J.Q., Nguyen M.H. (2016). Ethnic differences in incidence of hepatitis B surface antigen seroclearance in a real-life multicenter clinical cohort of 4737 patients with chronic hepatitis B infection. Aliment. Pharmacol. Ther..

[27] Tan A.T., Loggi E., Boni C., Chia A., Gehring A.J., Sastry K.S., Goh V., Fisicaro P., Andreone P., Brander C. (2008). Host ethnicity and virus genotype shape the hepatitis B virus-specific T-cell repertoire. J. Virol..

