# Supplementary material for: Caucasian Ethnicity, but Not Treatment Cessation Is Associated with HBsAg Loss Following Nucleos(t)ide Analogue-Induced HBeAg Seroconversion
Source: Viruses. 2019 Jul 26;11(8):687. doi: 10.3390/v11080687 (PMC6723144; doi:10.3390/v11080687)
Supplement: Supplementary file 1 [file viruses-11-00687-s001.pdf]

Supplementary Table 1: Propensity score adjusted Cox regression analysis to predict HBsAg loss following treatment-induced HBeAg seroconversion in the entire patient cohort. HR: Hazard Ratio. C.I.: Confidence Interval. Treatment cessation was investigated as a time-dependent covariate. Propensity scores were defined as the predicted probabilities calculated using a logistic regression model that assessed the correlation of both cirrhosis status and NA type with (1) Caucasian ethnicity; and (2) treatment cessation.

**Not adjusted** for propensity score			**Adjusted** for propensity score		
	p value	HR (95% C.I.)		*p* value	HR (95% C.I.)
Caucasian ethnicity (yes/no)	0.001	6.70 (2.26-21.40)	Caucasian ethnicity (yes/no)	0.001	6.95 (2.25-21.53)
			Propensity score (1)	0.794	0.70 (0.05-10.69)
Treatment cessation (yes/no; time-dependent)	0.372	1.45 (0.64-3.29)	Treatment cessation (yes/no)	0.211	1.76 (0.72-4.29)
			Propensity score (2)	0.375	0.42 (0.07-2.81)
Treatment cessation after at least 6 months consolidation (yes/no; time-dependent)	0.289	1.73(0.63-4.79)	Treatment cessation after at least 6 months consolidation treatment	0.185	2.08 (0.70-6.13)
			Propensity score (2)	0.361	0.42 (0.06-2.74)
Treatment cessation after at least 12 months consolidation (yes/no; time dependent)	0.525	1.52 (0.42-5.47)	Treatment cessation after at least 12 months consolidation treatment	0.387	1.82 (0.47-7.11)
			Propensity score (2)	0.374	0.42 (0.06-2.81)
